# Reconsolidation of a cocaine associated memory requires DNA methyltransferase activity in the basolateral amygdala

**DOI:** 10.1038/srep13327

**Published:** 2015-08-20

**Authors:** Hai-Shui Shi, Yi-Xiao Luo, Xi Yin, Hong-Hai Wu, Gai Xue, Xu-Hong Geng, Yan-Ning Hou

**Affiliations:** 1 Department of Pharmacy, Bethune International Peace Hospital of PLA, Shijiazhuang 050082, China; 2 Department of Biochemistry and Molecular Biology, Basic Medical College, Hebei Medical University, Shijiazhuang 050017, China; 3 Department of Pharmacology, Medical College of Hunan Normal University, Changsha 410013, China; 4 Department of Diagnosis Region of Function, Hebei Medical University Fourth Hospital, Hebei Medical University, Shijiazhuang, Hebei province, 050011.

## Abstract

Drug addiction is considered an aberrant form of learning, and drug-associated memories evoked by the presence of associated stimuli (drug context or drug-related cues) contribute to recurrent craving and reinstatement. Epigenetic changes mediated by DNA methyltransferase (DNMT) have been implicated in the reconsolidation of fear memory. Here, we investigated the role of DNMT activity in the reconsolidation of cocaine-associated memories. Rats were trained over 10 days to intravenously self-administer cocaine by nosepokes. Each injection was paired with a light/tone conditioned stimulus (CS). After acquisition of stable self-administration behaviour, rats underwent nosepoke extinction (10 d) followed by cue-induced reactivation and subsequent cue-induced and cocaine-priming + cue-induced reinstatement tests or subsequently tested to assess the strength of the cocaine-associated cue as a conditioned reinforcer to drive cocaine seeking behaviour. Bilateral intra-basolateral amygdala (BLA) infusion of the DNMT inhibitor5-azacytidine (5-AZA, 1 μg per side) immediately following reactivation decreased subsequent reinstatement induced by cues or cocaine priming as well as cue-maintained cocaine-seeking behaviour. In contrast, delayed intra-BLA infusion of 5-AZA 6 h after reactivation or 5-AZA infusion without reactivation had no effect on subsequent cue-induced reinstatement. These findings indicate that memory reconsolidation for a cocaine-paired stimulus depends critically on DNMT activity in the BLA.

The major hurdle for the successful treatment of cocaine addiction is relapse, which is often caused by craving-provoking cues that were previously paired with the rewarding effects of cocaine. It is now widely accepted that drug addiction involves the formation of aberrant memories termed “addiction memories” that play a critical role in the maintenance of learned addictive behaviours and relapse to drug seeking^1^,^2^,^3^,^4^,^5^. According to the memory reconsolidation hypothesis, memory traces are rendered labile after reactivation (retrieval) and must undergo a reconsolidation process to persist^6^,^7^,^8^,^9^. Studies from human and animal models of drug addiction suggest that pharmacological or non-pharmacological methods interfering with the reconsolidation of drug-associated memories can reduce or impair drug seeking behaviour^10^,^11^,^12^,^13^,^14^,^15^. Hence, elucidating the mechanism underlying the reconsolidation of drug memories will aid in the development of selective pharmacological therapies for prevention of drug relapse.

The basolateral amygdala (BLA) plays a critical role in the reconsolidation of drug-related associative memories^16^,^17^,^18^,^19^. Similar to the initial formation of long-term memories, reconsolidation requires *de novo* protein synthesis. Protein synthesis inhibitor administered immediately after reexposure to morphine-paired context blocked the reconsolidation of morphine reward memory^20^. Furthermore, knock-down of the transcription factor Zif268 in the BLA disrupted the reconsolidation of cued cocaine memory^10^,^21^. Moreover, BLA neurons are activated by the retrieval of cocaine-induced conditioned place preference (CPP), and intra-BLA infusion of lidocaine immediately after retrieval of cocaine-induced CPP diminishes subsequent CPP performance^22^, indicating that the BLA may be involved in the reconsolidation of cocaine reward memory. Our previous studies showed that the activity of cyclin-dependent kinase 5 (Cdk5), glycogen synthase kinase 3β(GSK-3β), or Rac1 in the BLA is required for the reconsolidation of cocaine cue memories^23^,^24^,^25^. Additionally, the activity of extracellular signal-regulated kinase (ERK) or protein kinase A (PKA) in the BLA is critical for the reconsolidation of cocaine memory as assessed by the instrumental paradigm^3^,^14^. Bilateral intra-BLA infusions of the PKA inhibitor Rp-cAMPS immediately following light/tone stimulus reactivation, but not delayed infusion 3 h after reactivation, decreased subsequent cue-induced reinstatement^3^. Moreover, infusion of Rp-cAMPS following no stimulus reactivation did not affect subsequent cue-induced reinstatement, indicating that memory reconsolidation for a cocaine-paired stimulus is retrieval-dependent and time limited, and critically depends upon PKA activity in the amygdala^3^. Another study showed that ERK activation in the BLA, but not in the nucleus accumbens core (NAc core), mediated the reconsolidation of Pavlovian cocaine memories^14^.

Although epigenetic mechanisms have been widely implicated in sustained synaptic plasticity underlying memory consolidation^26^,^27^,^28^,^29^,^30^,^31^, little is known of epigenetic regulation in memory reconsolidation. DNA methyltransferase (DNMT), an enzyme catalysing DNA methylation, is associated with transcriptional repression and has been implicated in hippocampus-and amygdala-dependent memory formation^32^. A recent study showed that intra-lateral amygdala (LA) infusion of the nucleoside analogue 5-AZA (1 μg in 0.5 μl/side) or the non-nucleoside DNMT inhibitorRG108 (1 μg in 0.5 μl/side) significantly impaired fear memory reconsolidation without facilitating extinction, suggesting that DNMT activity in the LA is critical for the reconsolidation of fear memory^33^. To date, no studies have addressed whether DNMT within the BLA in involved in appetitive memory reconsolidation. Hence, using post-retrieval BLA manipulations, the present experiments aim to examine the role of DNMT in the reconsolidation of cocaine-associated memories as assessed by the instrumental paradigm.

## Materials and Methods

### Subjects

Male Sprague Dawley rats (weighing 260–280 g on arrival) were housed in groups of five under controlled temperature (23 ± 2 °C) and humidity (50 ± 5%), and maintained on a 12-h light/dark cycle with access to food and water *ad* libitum. All of the rats were handled 3 minutes per day for 5 days before the surgeries. All animal procedures were performed in accordance with the National Institutes of Health Guide for the Care and Use of Laboratory Animals, and the experiments were approved by the Biomedical Ethics Committee for animal use and protection of Bethune International Peace Hospital of PLA. All the experiments were performed during the dark phase.

### Intracranial and intravenous surgery

Rats (weighing 300–320 g at the time of surgery) were anesthetized with sodium pentobarbital anesthesia (60  mg/kg, i.p.). Catheters were inserted into the right jugular vein with the tip terminating at the opening of the right atrium as described^15^,^34^,^35^. Guide cannulae (23 gauge; Plastics One, Roanoke, VA) were implanted bilaterally 1 mm above the BLA [anterior/posterior (AP), −2.9 mm from bregma; medial/lateral (ML), ±5.0 mm from bregma; dorsal/ventral (DV), −8.5 mm below the skull surface]^23^,^25^. The intravenous catheter was kept patent by infusion of 0.1 ml heparinized saline (30 USP heparin/saline; Hospira) every 2 d. The intracranial cannulae were kept patent by the insertion of obturators that were replaced daily. After surgery, rats were housed individually with food and water *ad libitum* until the end of experiments. Rats were allowed to recover for 5–7days before the start of the training and were weighed twice before training and daily once behavioral training commenced.

### Behavioral procedures

#### Intravenous cocaine self-administration training

The training method and conditions were modified from our previous studies^15^,^35^. The chambers (AniLab Software & Instruments, Ningbo, China) were equipped with two nosepoke operandi (AniLab Software & Instruments, Ningbo, China) located 9 cm above the floor of the chambers. A nosepoke in one (active) operandum led to cocaine infusion accompanied by a 5-s tone-light cue. Nosepokes in the other (inactive) operandum were also recorded but had no consequence. The modified cannula on the rat’s skull was connected to a liquid swivel (Instech, Plymouth Meeting, PA) with polyethylene-50 tubing protected by a metal spring and connected to a 10-ml syringe infusion pump. In brief, rats were trained to self-administer cocaine HCl intravenously (0.75 mg/kg/infusion) during three, 1-h, daily sessions separated by 5 min over 10 days. The sessions began at the onset of the dark cycle. A 1:1 fixed-ratio reinforcement schedule was used, with a 40-s timeout period after each infusion. Each session began with the illumination of a houselight that remained on for the entire 1-h session. Rats were deprived of food during the training sessions. The number of cocaine infusions was limited to 20 per hour. At the end of the training phase, rats were randomly divided into groups that were subjected to different protocols involving extinction, reactivation, BLA drug infusion, and (or) reinstatement tests (described below, Experiments 1–4). Groups were matched for their cocaine intake during training.

#### Extinction training

During the 3-h daily nosepoke extinction sessions (experiments 1–3), the stimulus light above the active nosepoke operandum was not illuminated. Nosepokes to either operandum had no programmed consequences (i.e. no cocaine infusion and no conditioned tone-light cue). 24 hours after cue-induced reinstatement test, during the 3-h daily cue extinction sessions (experiments 1–4), active nosepokes to the operandi triggered the same programmed consequences as in the cocaine self-administration training sessions (i.e., the conditioned tone-light cue) but without cocaine infusion. The rats were subjected to extinction training for 2 or 5 days until active operandum nosepoke frequency decreased below 20% of the mean response frequency during the last 3 days of cocaine self-administration for at least 2 consecutive days.

#### Reactivation of cocaine memory

A 15-min session to reactivate cocaine-associated memories commenced 24 hours after the lastnosepoke extinction session (for experiments 1 and 2) or 24 hours after the last self-administration session (for experiment 4). The retrieval conditions were the same as during cocaine self-administration training except that active nosepokes were not reinforced with cocaine.

#### Drug infusion

Infusions were performed immediately following the reactivation session (experiments 1 and 4) using a syringe pump with 10 μl Hamilton syringes connected to injection cannulae (28 gauge; Plastics One) via polyethylene tubing. Rats received a bilateral infusion of the DNMT inhibitor 5-AZA (1 μg/side at 0.25 μl/min for 2 min; Sigma-Aldrich) into the BLA while controls received an equal volume infusion of vehicle (0.5% DMSO). Injectors remained in place for 2 min following the infusion to allow for diffusion of the solution. As controls, other rats received 5-AZA or vehicle infusions 6 h after the reactivation session (experiment 2). Non- reactivated controls (experiment 3) were placed into the self-administration training chamber without presentations of the light/tone stimuli and then received infusions immediately following removal from the chamber. Rats were returned to their home cages following the infusion procedure.

#### Cue-induced reinstatement test (experiments 1–4)

Twenty-four hours after the intra-BLA 5-AZA or vehicle infusion, rats were tested for cue-induced reinstatement. For cue-induced reinstatement, rats were returned to the self-administration context and nosepokes to both operandi (active and inactive) recorded for 1 h. The testing conditions were the same as during cocaine self-administration training, with the exception that active nosepokes were not reinforced with cocaine. The test session began with illumination of the houselight, which remained on for the entire session. Nosepokes during the test sessions resulted in contingent presentations of the tone-light cue that was previously paired with cocaine infusions.

#### Cocaine + cue primed reinstatement test (experiments1–4)

Rats were injected with cocaine (10 mg/kg, i.p.) 5 minutes before transfer to the self-administration context for the reinstatement test. Nosepokes to both the active and inactive operandi were recorded. The test conditions were the same as during acquisition training with the exception that active nosepokes were not reinforced with cocaine, but with the delivery of cue (tone/light).

#### Histology

**Figure 1:**
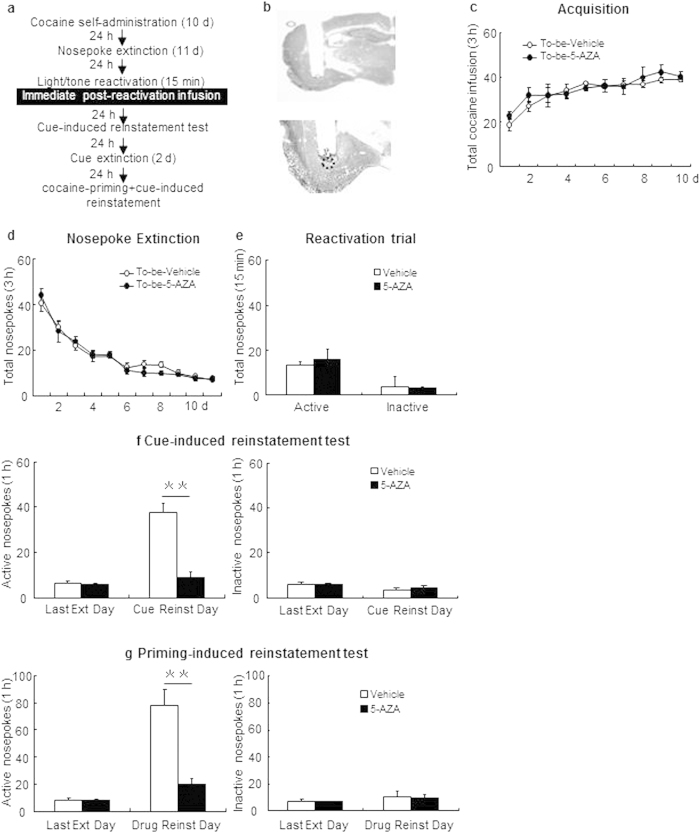
Immediate post-reactivation intra-BLA DNMT inhibition reduces subsequent cue-induced and cocaine-priming + cue-induced reinstatement. (**a**) Schematic representation of the experimental procedure. (**b**) Photomicrographs of representative cannula placements in BLA. (**c**) Total number of cocaine infusions across acquisition of cocaine self-administration sessions. (**d**) Total number of active nosepoke responses across nosepoke response extinction. (**e**) Nosepoke responses during reactivation trial. (**f**) Active (left) and inactive (right) nosepoke responses during the last extinction session of the nosepoke extinction sessions and the cue-induced reinstatement test. (**g**) Active (left) and inactive (right) nosepoke responses during the last extinction of the cue extinction sessions and the cocaine-priming + cue-induced reinstatement test. **Different from vehicle group, p < 0.01.

Histological slides were obtained from the experimental subjects. After behavioral tests were completed, all of the rats were anesthetized with sodium pentobarbital (100 mg/kg, i.p.) and transcardially perfused with paraformaldehyde. Cannula placements were assessed by Nissl’s staining of 40-μm thick coronal sections and examination under light microscopy^24^,^35^. The locations of representative cannula tips are shown in Fig. 1b.

### Specific experiments

#### Experiment 1: Effect of immediate post-reactivation intra-BLA DNMT inhibition on subsequent cue- and cocaine + cue primed reinstatement

We trained the rats to intravenously self-administer cocaine during three 1-h daily sessions over 10 days. Twenty-four hours after the last cocaine self-administration session, all rats in this experimental group received 10 d daily nosepoke extinction training in the original chamber. Twenty-four hours after the last nosepoke extinction session, all rats received a 15-min reactivation session induced by cocaine-associated cue reexposure in the training context. Immediately after the reactivation session, rats received bilateral intra-BLA infusion of 5-AZA or vehicle. Twenty-four hours later, all rats were tested for cue-induced reinstatement. After 2 days of daily cue extinction, rats were tested for priming-induced reinstatement (see Fig. 1a).

#### Experiment 2: Effect of delayed intra-BLA DNMT inhibition on subsequent cue- and cocaine + cue primed reinstatement

**Figure 2:**
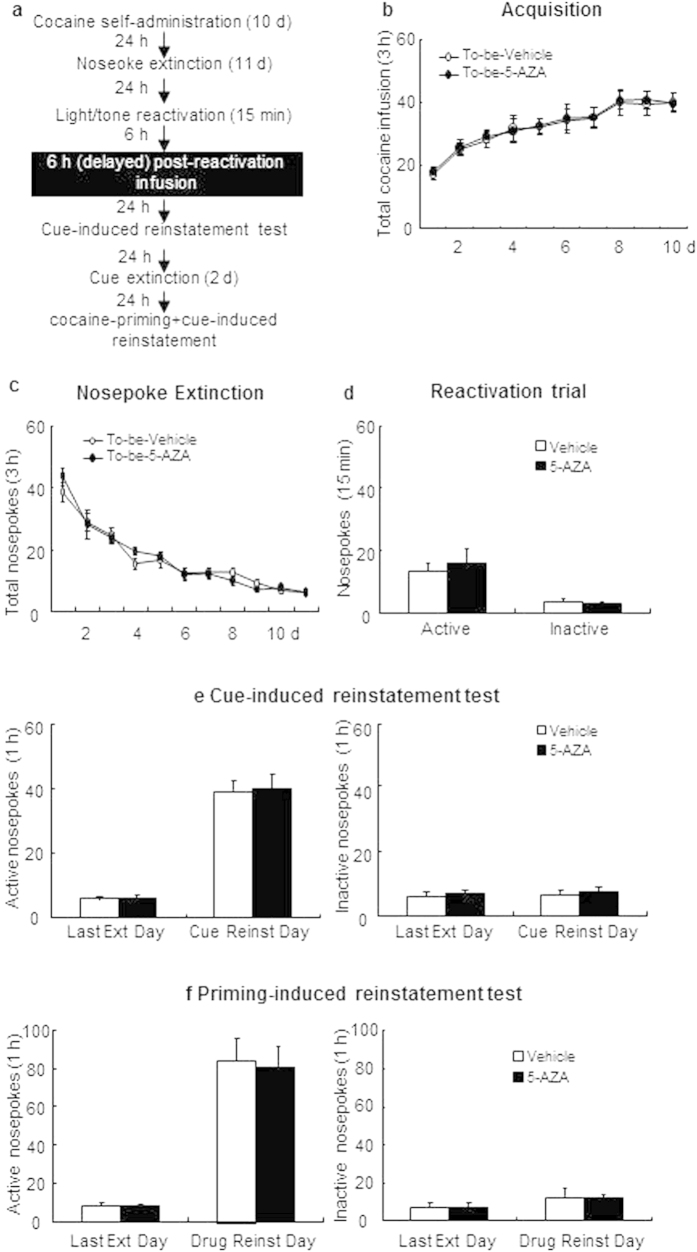
Intra-BLA DNMT inhibition 6 h following reactivation has no effects on subsequent cue-and cocaine-priming + cue-induced reinstatement. (**a**) Schematic representation of the experimental procedure. (**b**) Total number of infusions across acquisition of cocaine self-administration. (**c**) Total number of active nosepoke responses across response extinction. (**d**) Nosepoke responses during reactivation trial. (**e**) Active (left) and inactive (right) nosepoke responses during the last day of extinction and the cue-induced reinstatement test. (**f**) Active (left) and inactive (right) nosepoke responses during the last day of cue extinction and the cocaine-priming + cue-induced reinstatement test.

The experimental procedure for experiment 2 was identical to that of Experiment 1, except that the rats received bilateral intra-BLA infusion of 5-AZA or vehicle 6 hours after the 15-min reactivation session (see Fig. 2a).

#### Experiment 3: Effect of intra-BLA DNMT inhibition on subsequent cue-induced and cocaine + cue primed reinstatement in non-reactivated controls

**Figure 3:**
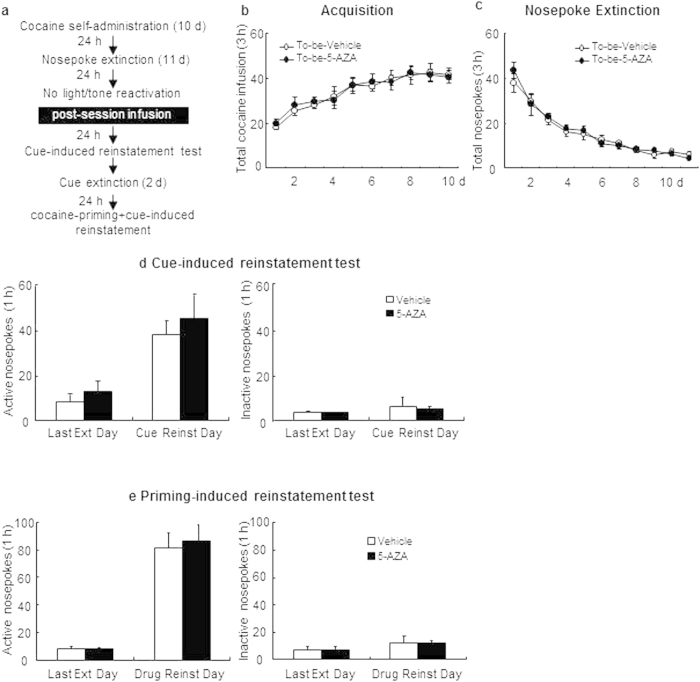
Intra-BLA DNMT inhibition has no effects on subsequent cue-and cocaine-priming + cue-induced reinstatement in non-reactivated controls. (**a**) Schematic representation of the experimental procedure. (**b**) Total number of infusions across acquisition of cocaine self-administration. (**c**) Total number of active nosepoke responses across extinction sessions. (**d**) Active (left) and inactive (right) nosepoke responses during the last day of extinction and the cue-induced reinstatement test. (**e**) Active (left) and inactive (right) nosepoke responses during the last day of cue extinction and the cocaine-priming + cue-induced reinstatement test.

The experimental procedure for experiment 3 was identical to that of experiment 1, except that the rats received intra-BLA infusion immediately after a 15 min no-reactivation session that is similar with the nosepoke extinction in the training chamber and that the rats received bilateral intra-BLA infusion of 5-AZA or vehicle 24 hours after the last nosepoke extinction session with no intervening reactivation session (see Fig. 3a).

#### Experiment 4: Effect of immediate post-reactivation intra-BLA DNMT inhibition on subsequent conditioned cue-maintained cocaine seeking behavior

**Figure 4:**
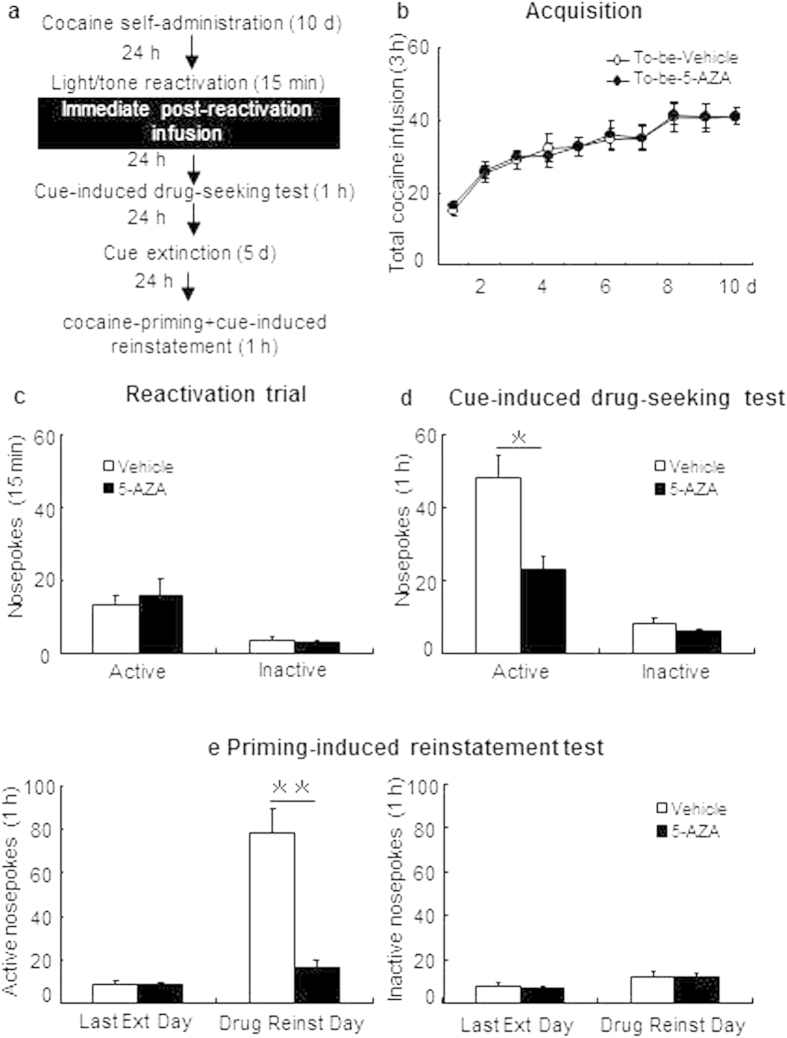
Immediate post-reactivation intra-BLA 5-AZA infusion decreases the subsequent cue-maintained cocaine seeking behaviors. (**a**) Schematic representation of the experimental procedure. (**b**) Total number of infusions across acquisition of cocaine self-administration. (**c**) Nose-poke responses during the reactivation trial. (**d**) Nose-poke responses during the conditioned reinforcement test. (**e**) Active (left) and inactive (right) nosepoke responses during the last day of extinction and the cocaine-priming + cue-induced reinstatement test. *Different from vehicle group, p < 0.05; **Different from vehicle group, p < 0.01.

We trained rats to intravenously self-administer cocaine during three 1-h daily sessions over 10 days. To test the effects of disruption of the cocaine-cue memory on the cue-maintained cocaine-seeking behavior, we omitted the nosepoke extinction sessions in this experiment. Twenty-four hours after the last cocaine self-administration session, all rats received a 15-min reactivation session induced by cocaine-associated cue reexposure. Immediately after the reactivation session, rats received bilateral intra-BLA infusion of 5-AZA or vehicle. Twenty-four hours later, all rats were tested for cue-induced reinstatement. After 5 days of daily cue extinction, rats were subsequently tested for cocaine priming-induced reinstatement (see Fig. 4a).

### Statistical analysis

We reported the results as mean ± SEM and analyzed the data by two-way/repeated measures ANOVAs with treatment condition (5-AZA vs. vehicle) as the between-subjects factor and test condition (last nosepoke extinction day vs. cue-induced cocaine or last cue extinction day priming-induced reinstatement test) as the within-subjects factor for each experiment (see results). The active and inactive poking numbers were analyzed separately. Turkey’s post-hoc tests were used for specific pair-wise comparisons in cases of significant main effects and interactions (p < 0.05, two-tailed) from the two-way ANOVAs.

## Results

### Experiment 1: Immediate post-reactivation intra-BLA DNMT inhibition reduces subsequent reinstatement of cocaine-seeking behavior

In experiment 1, we used two groups of rats to test the effect of intra-BLA DNMT inhibition on cue- and cocaine-priming + cue-induced reinstatement of cocaine seeking behaviors (Fig. 1a). All of the cannula placements were within the boundaries of the BLA as revealed by post-experimental histology (Fig. 1b). The number of cocaine infusions was analyzed by repeated measures (rm)-ANOVA, with the treatment condition as the between-subjects factor and the training day as within-subject factor. Acquisition of cocaine self-administration did not differ between rats (subsequently) infused with 5-AZA (N = 9) and those infused with vehicle (N = 7) as indicated by the total number of cocaine infusions [main effect of the training day: F(9,126) = 15.941, p < 0.01; main effect of the treatment condition: F(1,14) = 0.598, p = 0.452; interaction of training day × treatment condition: F(9,126) = 0.296, p = 0.975; Fig. 1c]. In addition, there was no difference in nosepoke extinction rate as revealed by the daily numbers of active (reinforced) and inactive (non-reinforced) nosepokes over the 10-day extinction trial by rm-ANOVA with the treatment conditions as the between-subjects factor and the extinction day as within-subject factor [main effect of the extinction day: F(10,140) = 55.999, p = 0.000; main effect of the treatment condition: F(1,14) = 0.257, p = 0.620; interaction of extinction day × treatment condition: F(10,140) = 0.815, p = 0.614; Fig. 1d]. For the reactivation trial, there were no group differences in the numbers of active [F(1,14) = 0.126 p = 0.728] and inactive nosepokes [F(1,14) = 1.717 p = 0.686; Fig. 1e]. There was, however, a significant difference of active nosepokes between treatment groups (5-AZA vs. vehicle) in the reinstatement tests. Repeated measures ANOVA (with the treatment condition as a between-subjects factor and test condition as a within-subject factor) revealed significant cue-induced reinstatement test × drug treatment condition interaction for number of active side nosepokes [main effect of the test condition: F(1, 14) = 53.072, p < 0.01; main effect of the treatment condition: F(1,14) = 46.314, p < 0.01; interaction of extinction day × treatment condition: F(1,14) = 36.518, p < 0.01; Fig. 1f left column], but not inactive side nosepokes [main effect of the test condition: F(1,14) = 3.156, p = 0.097; main effect of the treatment condition: F(1,14) = 0.382, p = 0.546; interaction of extinction day × treatment condition: F(1,14) = 0.504, p = 0.490; Fig. 1f right column]. In addition, there is a significant cocaine-priming + cue-induced reinstatement test × treatment condition interaction for active side nosepokes [main effect of the test condition: F(1,14) = 50.644, p < 0.01; main effect of the treatment condition: F(1,14) = 27.263, p < 0.01; interaction of test condition × treatment condition: F(1,14) = 32.383, p < 0.01]; Fig. 1g left column], but not inactive side nosepokes [main effect of the test condition: F(1,14) = 1.844, p = 0.196; main effect of the treatment condition: F(1,14) = 0.026, p = 0.875; interaction of test condition × treatment condition: F(1,14) = 0.005, p = 0.944; Fig. 1g right column]. Results of this experiment indicate that inhibition of DNMT activity by intra-BLA infusion of 5-AZA suppressed cocaine-associated cue- and cocaine-priming + cue-induced reinstatement.

### Experiment 2: Delayed intra-BLADNMT inhibition following reactivation has no effect on subsequent reinstatement of cocaine-seeking behavior

In experiment 2, we aimed to identify the temporal window for this effect of intra-BLA DNMT blockade on reconsolidation of cocaine memory (Fig. 2a). During acquisition of cocaine self-administration, there is no difference in total infusion between the rats that would be infusedwith5-AZA (N = 6) and those infused with vehicle (N = 6) [main effect of the training day: F(9,90) = 20.645, p < 0.01; main effect of the treatment condition: F(1,10) = 0.100, p = 0.758; interaction of training day × treatment condition: F(9,90) = 0.095, p = 0.999; Fig. 2b]. Similarly, there were no group differences in the rate of extinction as revealed by active [main effect of extinction day: F(10,100) = 37.157, p < 0.01; main effect of the treatment condition: F(1,10) = 0.204, p = 0.661; interaction of extinction day × treatment condition: F(10,100) = 0.283, p = 0.984; Fig. 2c] and inactive nosepokes (data not shown). For the reactivation trial, there were no group differences in the numbers of active nosepokes [F(1,10) = 0.370, p = 0.556; Fig. 2d] and inactive nosepokes [F(1,10) = 0.077, p = 0.787; Fig. 2d]. In contrast to results from Experiment 1, however, intra-BLA infusion of 5-AZA 6 hours following reactivation had no effects on subsequent cue-induced reinstatement of cocaine-seeking behaviors responded to active nosepokes [main effect of the test condition: F(1,10) = 75.762, p < 0.01; main effect of the treatment condition: F(1,10) = 0.057, p = 0.816; interaction of test condition × treatment condition: F(1,10) = 0.008 p = 0.932; Fig. 2e, left column] and inactive nosepokes [main effect of the test condition: F(1,10) = 0.007, p = 0.935; main effect of the treatment condition: F(1,10) = 0.356, p = 0.564; interaction of test condition × treatment condition: F(1,10) = 0.062; p = 0.808; Fig. 2e, right column], as well as on the cocaine-priming + cue-induced reinstatement of response to active nosepokes [main effect of the test condition: F(1,10) = 58.950, p < 0.01; main effect of the treatment condition: F(1,10) = 0.057, p = 0.816; interaction of test condition × treatment condition: F(1,10) = 0.044, p = 0.839; Fig. 2f, left column] and inactive nosepokes [main effect of the test condition: F(1,10) = 0.059, p = 0.813; main effect of the treatment condition: F(1,10) = 0.298; p = 0.597; interaction of test condition × treatment condition: F(1,10) = 0.044, p = 0.839; Fig. 2f, right column]. Therefore, the effect of 5-AZA is time limited in that inhibition of DNMT must occur within 6 hours of reactivation to block reinstatement.

### Experiment 3: Intra-BLA DNMT inhibition with no reactivation has no effect on subsequent reinstatement cocaine-seeking behavior

In experiment 3, we examined whether the function of BLA DNMT in reconsolidation of cocaine memory is reactivation dependent. After acquisition of cocaine self-administration and extinction as described for Experiments 1, rats received intra-BLA infusion of either 5-AZA or vehicle immediately after a 15-min exposure to the training chamber but without light/tone cue delivery (Fig. 3a). There was no difference in either acquisition of cocaine self-administration sessions [main effect of the training day: F(9,90) = 21.595, p < 0.01; main effect of the treatment condition: F(1,10) = 0.269, p = 0.615; interaction of training day × treatment condition: F(9,90) = 0.174, p = 0.996; Fig. 3b] or the nosepoke extinction rates [main effect of the extinction day: F(10,100) = 44.087, p < 0.01; main effect of the treatment condition: F(1,19) = 0.379, p = 0.552; interaction of extinction day × treatment condition: F(10,100) = 0.475 P = 0.903; Fig. 3c] between rats that would be infused with 5-AZA (N = 6) and those infused with vehicle (N = 6). In the cue-induced reinstatement, there was no difference in either active nosepokes [main effect of the test condition: F(1,10) = 47.382, p < 0.01; main effect of the treatment condition: F(1,10) = 1.027, p = 0.335; interaction of test condition × treatment condition: F(1,10) = 0.000, p = 0.985; Fig. 3d, left column] or inactive nosepokes [main effect of the test condition: F(1,10) = 2.918, p = 0.118; main effect of the treatment condition: F(1,10) = 0.096, p = 0.763; interaction of test condition × treatment condition: F(1,10) = 0.031, p = 0.863; Fig. 3d, right column] between the groups, as well as in the subsequent cocaine-priming + cue-induced reinstatement test, there was no difference in either active nosepokes [main effect of the test condition: F(1,10) = 69.208, p < 0.01; main effect of the treatment condition: F(1,10) = 0.068, p = 0.800; interaction of test condition × treatment condition: F(1,10) = 0.076, p = 0.788; Fig. 3e, left column] or inactive nosepokes [main effect of the test condition: F(1,10) = 0.073, p = 0.793; main effect of the treatment condition: F(1,10) = 0.032, p = 0.862; interaction of test condition × treatment condition: F(1,10) = 0.146, p = 0.710; Fig. 3e, right column]. In summary, results from this experiment indicate that the effect of 5-AZAobserved in experiment 1 depends on the light/tone stimulus and that infusion of 5-AZA into the BLA alone had no effects on the following cue-induced or cocaine-priming + cue-induced reinstatement.

### Experiment 4: Immediate post-reactivation intra-BLA DNMT inhibition reduces subsequent conditioned cue-maintained cocaine seeking behavior

In experiment 4, we investigated whether immediate post-reactivation intra-BLA DNMT inhibition reduces subsequent cue-maintained drug seeking behaviors. All rats were trained for intravenous cocaine self-administration as described but received 15-min light/tone reactivation 24 h after acquisition without intervening extinction and then immediately subjected to intra-BLA infusion of AZA-5 or vehicle (Fig. 4a). Acquisition of cocaine self-administration did not differ between the rats that would be infusedwith5-AZA (N = 9) versus vehicle (N = 9) following reactivation as there were no differences in the total number of cocaine infusions [main effect of the training day: F(9,144) = 22.042, p < 0.01; main effect of the treatment condition: F(1,16) = 0.039, p = 0.846; interaction of training day × treatment condition: F(9,144) = 0.076, p = 0.997; Fig. 4b], active nosepokes, or inactive nosepokes (data not shown). For the reactivation trial, there were no group differences in the numbers of active and inactive nosepokes [F(1,16) = 0.138, p = 0.715; F(1,16) = 0.222, p = 0.188 respectively; Fig. 4c]. However, there were significant group effects of in cue-induced reinstatement for active nosepokes [F(1,16) = 4.957, p = 0.022; Fig. 4d], but not inactive nosepokes [F(1,16) = 0.016, p = 0.984; Fig. 4d]. In addition, there is a significant cocaine-priming + cue-induced reinstatement test × treatment condition interaction for active nosepokes [main effect of the test condition: F(1,16) = 34.869, p < 0.01; main effect of the treatment condition: F(1,16) = 24.61, p < 0.01; interaction of test condition × treatment condition: F (1, 16) = 21.733, p < 0.01; Fig. 4e, left column], but not inactive nosepokes [main effect of the test condition: F(1,16) = 3.894, p = 0.066; main effect of the treatment condition: F(1,16) = 0.126, p = 0.727; interaction of test condition × treatment condition: F(1,16) = 0.073, p = 0.790; Fig. 4e, right column]. These findings indicate that immediate post-reactivation intra-BLA DNMT inhibition reduced conditioned cue-maintained cocaine-seeking behaviors and that this inhibitory effect was resistant to subsequent cocaine priming. This inhibitory effect of intra-BLA DNMT inhibition on subsequent reinstatement of cocaine seeking is due to disruption of cocaine-associated memory reconsolidation.

## Discussion

We investigated the contribution of epigenetic processes mediated by DNMT in the BLA for the reconsolidation of cocaine reward memory. Immediate, but not delayed (6 hours), intra-BLA infusion of the DNMT inhibitor 5-AZA following reactivation by presentation of a stimulus previously paired with cocaine self-administration (a light/tone CS) disrupted the ability of this CS to reinstate cocaine seeking or to serve as a conditioned reinforcer for cue-maintained cocaine seeking. On the other hand, intra-BLA 5-AZA infusion in non-reactivated rats has no effects on subsequent cue-induced reinstatement. These findings indicate that inhibition of DNMT in the BLA attenuates reinstatement of cocaine-seeking behaviour as well as the maintenance of cocaine seeking by disrupting the reconsolidation of cocaine-associated memories.

Drug-associated cue-induced relapse is the major problem for successful treatment of cocaine addiction^36^,^37^,^38^,^39^. Neutral environmental cues acquire conditioned reinforcing properties through repeated pairing with the rewarding effects of abused drugs to maintain the drug seeking and precipitate reinstatement^36^,^39^,^40^,^41^. Studies from animal models of relapse have suggested that interfering with reconsolidation by pharmacological or non-pharmacological manipulations holds great potential for persistently inhibiting cue-induced reinstatement^3^,^10^,^11^,^14^,^15^,^21^,^42^.

That 5-AZA blocking reinstatement of cocaine-seeking behaviour as well as the maintenance of cocaine seeking by disrupting the reconsolidation of cocaine-associated memories is supported by the following lines of evidence. First, we found that immediate post-reactivation BLA infusions of 5-AZAblocked the cue-induced reinstatement and that this inhibitory effect on cue-induced reinstatement wasresistant to noncontingent re-exposure to cocaine priming, indicating that inhibitory effects on subsequent reinstatement tests was not produced by temporarily inhibiting retrieval of cocaine cue memory. Second, these infusions had no effect on subsequent cue-induced reinstatement in rats that did not undergo cue reactivation, indicating that the inhibitory effect on cue-induced reinstatement of cocaine-seeking is retrieval-dependent. Third, intra-BLA infusions of 5-AZA6h after cue reactivation had no effect on cocaine-associated cue-induced reinstatement of cocaine seeking, demonstrating a critical post-retrieval time window during which DNMT is required for reconsolidation, consistent with previous studies investigating the reconsolidation of drug-memory using either self-administration or CPP paradigms^3^,^11^,^14^,^18^,^24^,^25^. However, Sanchez *et al*. found that disruption of cocaine-associated stimulus only decreased the cue-induced reinstatement, but has no effect on the cocaine-priming + cue-induced reinstatement, which is different from the findings in the current study 3. These contrary findings may result from the different experimental conditions, including the memory activation context (novel or original training chamber), performed cue extinction sessions or not before cocaine-priming reinstatement test, delivery of light/tone stimulus during the cocaine-priming reinstatement test. Since cocaine-associated cues acquired motivational value through repeated pairing with cocaine infusion during self-administration sessions, manipulations that disrupt the association between cues and cocaine reward (e.g. interfering with consolidation or reconsolidation of drug memory) diminished the incentive/motivational properties of the conditioned cues^3^,^21^,^43^. If the conditioned reinforcement of drug-associated cues is diminished by disrupting the reconsolidation of cocaine cue memory induced by intra-BLA DNMT inhibition, the capacity of cocaine-associated cues to maintain drug-seeking behavior should be weakened. Therefore, in experiment 4 we tested the ability of the cocaine-paired cue to maintain originally learned drug-seeking behavior during extinction, which may also reflect the conditioned reinforcement properties of cocaine-paired cues. Finally, we found that immediate intra-BLA 5-AZA infusion following cue-induced retrieval impaired the ability of the cocaine-paired cue to maintain cocaine-seeking behavior, suggesting that this manipulation devalued the conditioned reinforcing effects by disrupting the conditioned association between the cocaine-paired cue and the reinforcing effect of self-administered cocaine. Together, post-reactivation DNMT inhibition in the BLA reduced subsequent cue-induced cocaine seeking behavior. This inhibitory effect is specific to the reactivated memories and is time-limited, indicating that DNMT inhibition in the BLA targets the reconsolidation of cocaine memory.

It has been demonstrated that de novo transcription is required for memory reconsolidation^8^,^44^,^45^,^46^,^47^. Epigenetic mechanisms including chromatin restructuring and DNA methylation regulate transcription^48^,^49^,^50^,^51^. It has been reported that the methylation of cytosine residues on DNA catalyzed by DNMTs causes the chromatin structure to compact, leading to transcriptional suppression^32^,^49^,^52^,^53^,^54^. Moreover, high levels of DNMT mRNA are expressed by adult neurons^32^,^33^,^48^,^55^,^56^,^57^. Therefore, dynamic regulation of DNA methylation is likely critical for neuronal function, including synaptic plasticity. Considering that de novo transcription is required for memory reconsolidation and that DNA methylation has been associated with transcriptional repression, DNMT inhibition should positively regulate memory reconsolidation by promoting transcription. However, several studies reported that DNMT inhibition negatively regulated both memory consolidation and reconsolidation^30^,^54^,^58^. Previous studies have shown that DNMT activity is required for hippocampal-dependent or amygdala-dependent memory consolidation^30^,^32^,^54^,^59^. Recently, DNMT activity in the lateral nucleus of the amygdala was suggested to be critical for reconsolidation of fear memory. Inhibition of DNMT activity in the LA significantly impaired fear memory reconsolidation in a time-limited and retrieval-dependent manner^33^. During the reconsolidation process, the labile memory needs to be restabilized, which requires new gene transcription. DNMT inhibition impairs reconsolidation of fear memory may induced by the reason that gene transcription needs to be shut down and the DNA return to a more repressed state for the memory restabilization. DNMT inhibition may block the re-repression of gene transcription leading to memory may remain labile and become impaired. There are several DNMT inhibitors available, including the nucleoside analogue 5-AZAandthe non-nucleoside inhibitor RG108. 5-AZA is considered an S-phase-specific nucleoside analogue that inhibits DNA methylation during DNA replication^32^, by which 5-AZA can effectively modulate DNA methylation in the hippocampus and hippocampal- and amygdala-dependent behaviors^48^,^54^,^60^. Our present data are consistent with the role of amygdalar DNMT modulation in memory reconsolidation of auditory fear, indicating that a conserved mnemonic process governing both appetitive and aversive stimulus memories is possible^16^. In the present studies, we found that DNMT inhibition by 5-AZAin the BLA immediately after reactivation resulted in a similar retrieval-dependent deficit in the reconsolidation of cocaine-associated memories. However, these results should be interpreted with caution as 5-AZA was infused immediately after extinction conditions (i.e., a cue with no cocaine reinforcement) and so may have affected synaptoplastic processes related to extinction. Theoretically, DNMT inhibition should promote transcription to enhance the consolidation of extinction memory. Strengthened extinction memory renders the original cocaine-associated memory less susceptible to reinstatement, spontaneous recovery, and renewal^61^,^62^,^63^. However, the inhibitory effects of immediately after memory reactivation DMNT inhibition in the BLA on the cocaine seeking can’t interpreted by the extinction enhancement in the current study, owing to that we found that this inhibitory effects was resistant to reexposure to the noncontingent cocaine priming. Another limitation is that lack of the biological evidence for changes in DMNT activity during/after reactivation of cocaine-associated memory. Further experiments are required to determine how 5-AZA affects the methylation of genes in the BLA following retrieval of cocaine-associated memories. Given that the epigenetic regulation of gene expression is complex, including DNA methylation, histone acetylation, chromatin remodeling, and non-coding RNAs regulation^49^,^64^,^65^,^66^,^67^, the underlying mechanism of DNA methylation during memory reconsolidation may involve multiple epigenetic mechanisms, such as histone modification, which should be elucidated in the future studies.

## Concluding Remarks

In summary, the results of the present work provide new evidence that DNA methylation regulates there consolidation of cocaine-associated memories within the BLA. This study is the first of which we are aware to examine the role of epigenetic mechanisms, specifically DNA methylation, in the reconsolidation of drug reward memory and these results may enhance our understanding of the cellular and molecular mechanisms of drug memory reconsolidation within the BLA.

## Additional Information

**How to cite this article**: Shi, H.-S. *et al*. Reconsolidation of a cocaine associated memory requires DNA methyltransferase activity in the basolateral amygdala. *Sci. Rep*. **5**, 13327; doi: 10.1038/srep13327 (2015).
